# Applying Bioinformatic Platforms, In Vitro, and In Vivo Functional Assays in the Characterization of Genetic Variants in the GH/IGF Pathway Affecting Growth and Development

**DOI:** 10.3390/cells10082063

**Published:** 2021-08-12

**Authors:** Sabina Domené, Paula A. Scaglia, Mariana L. Gutiérrez, Horacio M. Domené

**Affiliations:** Centro de Investigaciones Endocrinológicas ‘Dr César Bergadá’, (CEDIE) CONICET, FEI, División de Endocrinología, Hospital de Niños Ricardo Gutiérrez), Buenos Aires C1425EFD, Argentina; sdomene@cedie.org.ar (S.D.); pscaglia@cedie.org.ar (P.A.S.); marianagutierrez@cedie.org.ar (M.L.G.)

**Keywords:** bioinformatics, functional assays, growth and development, GH-IGF axis, short stature, next-generation sequencing

## Abstract

Heritability accounts for over 80% of adult human height, indicating that genetic variability is the main determinant of stature. The rapid technological development of Next-Generation Sequencing (NGS), particularly Whole Exome Sequencing (WES), has resulted in the characterization of several genetic conditions affecting growth and development. The greatest challenge of NGS remains the high number of candidate variants identified. In silico bioinformatic tools represent the first approach for classifying these variants. However, solving the complicated problem of variant interpretation requires the use of experimental approaches such as in vitro and, when needed, in vivo functional assays. In this review, we will discuss a rational approach to apply to the gene variants identified in children with growth and developmental defects including: (i) bioinformatic tools; (ii) in silico modeling tools; (iii) in vitro functional assays; and (iv) the development of in vivo models. While bioinformatic tools are useful for a preliminary selection of potentially pathogenic variants, in vitro—and sometimes also in vivo—functional assays are further required to unequivocally determine the pathogenicity of a novel genetic variant. This long, time-consuming, and expensive process is the only scientifically proven method to determine causality between a genetic variant and a human genetic disease.

## 1. Introduction

Heritability accounts for more than 80% of adult human height [1], indicating that genetic variability is the main determinant of stature. Over 700 common variants (single nucleotide polymorphisms, SNPs) across more than 400 loci have been independently associated with height [2].

**Figure 1:**
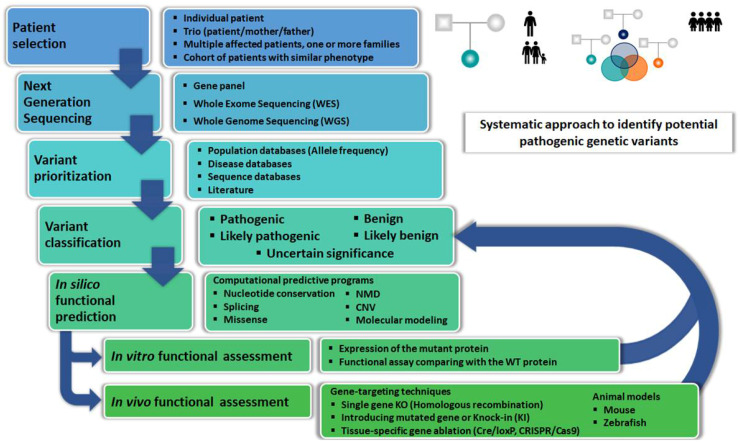
Systematic approach to identify potential pathogenic genetic variants. Flowchart for a systematic approach to select and characterize potential pathogenic genetic variants in children with growth and development disorders. The selection of patient(s) can be directed to individual patients, a trio including the patient and his/her parents, multiple families presenting several affected index cases, or a cohort of patients sharing a similar phenotype. Next-Generation Sequencing (NGS) can be performed using gene panels, Whole Exome Sequencing (WES), or Whole Genome Sequencing (WGS). For variant(s) prioritization, population, disease, and sequence databases are used. Systematic literature review is also required. Variants are then classified according to ACMG criteria as pathogenic, likely pathogenic, of uncertain significance, likely benign, or benign. In silico functional assessment is based upon several computational predictive programs. Finally, to unequivocally determine the pathogenicity of a novel genetic variant, further in vitro functional assays and sometimes in vivo animal models are required.

The GH/IGF axis plays an important role in pre and postnatal growth. In 1981, Phillips III and their colleagues described the first molecular characterization in this axis: the complete *GH1* gene deletion, resulting in familial isolated complete GH deficiency [3]. Since then, molecular defects in more than 48 different genes have been described all along the GH/IGF axis [4,5]. These defects result in alteration of GH synthesis and secretion, impairment on GH action, in IGF1 synthesis, transport and action, and in IGF2 synthesis. Most of these molecular defects were discovered by the candidate gene approach using clinical data and biochemical profiles to select the more likely candidate gene(s) to be studied. In recent years, the candidate gene approach to the molecular diagnosis of genetic diseases has been replaced with copy number variation (CNV) analysis based on chromosomal microarrays, sequencing of a genetic panel (including several candidate genes), whole-exome sequencing (WES), and whole-genome sequencing (WGS) technologies (Figure 1). The diagnostic effectiveness to detect a genetic defect as etiology of short stature using these technologies is about 25–40% [6]. Since 2012, with the extended use of these techniques, novel genetic clinical conditions have been elucidated in short patients where clinical and biochemical data did not suggest an obvious candidate gene. In addition, this novel approach has also detected molecular defects affecting growth in genes beyond the GH/IGF axis.

One of the greatest challenges of WES and WGS remains the high number of candidate variants identified. Most of these are novel or ultra-rare variants that need to be classified according to their pathogenicity. In silico bioinformatic tools are essential for a first approach to classifying these candidate variants based on evolutionary conservation, type of amino acid change, position within a functional domain, allele frequency in the general population, and other parameters [7]. The American College of Medical Genetics and Genomics (ACMG) recommendations for the interpretation of sequence variants are a useful tool for the classification of genetic variants as pathogenic, likely pathogenic, uncertain significance, likely benign, and benign [8]. However, these bioinformatic tools are not a comprehensive solution to solving the complicated problem of variant interpretation and require to be complemented with the use of experimental approaches such as in vitro and when needed, in vivo functional assays in order to determine the true nature of the variant. Even though most nonsense or frameshift variants in genes are thought to be loss-of-function (LOF) alleles due to nonsense-mediated decay (NMD) of the encoded transcript, truncating mutations found in the last exons escape this process and may function as benign or gain-of-function (GOF) alleles [9]. Predicting the effects of a missense allele is a very challenging task, since it can result in a number of different genetic scenarios as first described by Herman Muller in the 1930s (i.e., amorph, hypomorph, hypermorph, antimorph, neomorph, or isomorph) [10]. The ability to determine the functionality and pathogenicity of variants will not only benefit the patient under study, by providing accurate molecular diagnosis, and consequently provide adequate treatment and clinical follow-up for the early detection of potential comorbidities, but will also have broader impacts on both translational and basic scientific research.

The short stature phenotype may be either proportionate or disproportionate, severe, or mild, and with a prenatal or postnatal origin [11]. Clinical evaluation and biochemical and radiological assessment are very important to determine the preliminary diagnosis. When a genetic etiology is suspected (i.e., rasopathies, isolated GH deficiency, skeletal dysplasia), genetic panels can be used [12,13]. In those cases, in which no obvious candidate genes are suspected, WES is usually the indicated technique. For individual patients, it has been extremely useful to perform WES to both the proband and their parents (trio). Extended family pedigrees also help to identify the pattern of inheritance (recessive, dominant, or X-linked) additional aiding in the selection of candidate genetic variants in the WES. Similar approaches can be used in the study of several families presenting a similar phenotype. Finally, in cohort studies sharing a similar phenotype, WES is useful to select potential pathogenic variants found in a representative fraction of the selected cohort.

Inherited large germline rearrangements such as CNVs have been demonstrated to cause a significant number of human hereditary disorders by different molecular mechanisms, including dosage effects, gene disruption and position effects [14]. Recent studies have identified pathogenic or probably pathogenic CNVs in a significant proportion of children with short stature of unknown origin, particularly in syndromic patients with or without neurodevelopmental disorders [15,16,17,18,19,20]. Thus, the analysis of CNVs remains a very important step in a rational approach to the genomic diagnosis of growth disorders in children. Array-based genomic copy number analyses, including comparative genomic hybridization array (aCGH) and SNP-arrays, and Multiplex Ligation Probe Amplification (MLPA) are the gold standard methods for large CNV detection. Even if NGS techniques are very efficient for the detection of small deletions or insertions and single nucleotide variants (SNVs), large CNV detection from NGS data is quite challenging mainly due to short read length, GC content and variable efficiency of the capture process [21]. However, as the use of NGS becomes more readily available for the evaluation of short stature patients, applying bioinformatic algorithms to NGS data will become a rapid and cost-effective method to screen for the presence of CNVs and select the best candidates for confirmation using further studies such as MLPA or aCGH/SNP array. The main purpose of genetic testing in children with short stature is to identify rare variants responsible for monogenic defects. A molecular diagnosis is relevant for the patient and their family because it not only results in the end of the diagnostic workflow but could also result in the selection of the most convenient therapeutic approach. In addition, a genetic diagnosis suggests a rational follow-up for the early detection of medical comorbidities associated with the characterized genetic condition. Although there is not a general consensus on the selection criteria concerning which patients would benefit from genetic testing [4,22,23,24,25], it has been proposed that there are several factors that could increase the possibility to detect a monogenic cause of short stature: severe GH deficiency, multiple pituitary hormone deficiency (MPHD), unequivocal GH insensitivity, small for gestational age (SGA) without catch-up growth, additional congenital anomalies or dysmorphic features, evidence of skeletal dysplasia, associated intellectual disability, microcephaly, and severe growth retardation (height below −3.0 SD) [12]. In addition to classical Mendelian genetic defects, it is also important to bear in mind that growth and development can also be affected by an accumulative effect of multiple common variants. In fact, genetic studies of adult height have shown that a genetic score composed of common variants (“polygenic risk score”) was associated with adult height at the extremes of the population distribution [26].

In this review, we will discuss a rational approach to apply to the gene variants identified in children with growth and developmental defects including: (i) bioinformatic tools to characterize potential pathogenicity; (ii) in silico modeling tools to predict conformational distortions; (iii) in vitro functional assays to determine their pathogenicity; and (iv) the development of in vivo models to determine the impact of a given variant on the whole organism.

## 2. Bioinformatic Tools

NGS has facilitated the collection and analysis of a large amount of genomic, transcriptomic, proteomic, and metabolomic data from different organisms that have allowed predictions on the regulation of expression, transcription, translation, structure, and mechanisms of action of proteins. Although the information available in the databases is growing every day, all bioinformatic tools continue to be constantly modified to improve performance that leads to more accurate predictions regarding protein functionality. Some examples of frequently used predictive algorithms used for variant pathogenicity inference and for CNV detection using NGS data are listed in Table S1: In silico predictive algorithms frequently used for variant pathogenicity inference and for CNV de-tection using NGS data.

The process of defining the potential role of a certain variant as disease-causing over many others obtained in an NGS study is frequently known as variant prioritization. The certainty with which any given sequence variant may be considered as clinically relevant falls within a spectrum, ranging from almost certain to be disease-causing to almost certainly not related to the phenotype [8]. In this process, many aspects should be considered. First, as a quality control step, only variants fulfilling the adequate criteria should be included for further analysis (usually 20× minimum depth, appropriate genotype-assessment quality, without strand bias). Once variants with adequate quality have been selected, the analysis of several of their characteristics will allow us to select the most promising candidates to explain the observed phenotype. Major features considered are variant frequency in the general population and the availability of reports in the literature or in disease-specific databases linking that variant to a certain phenotype. When diagnosing rare diseases, a high allele frequency in the general population (usually an arbitrary threshold >5%) for a particular variant classifies it as benign. After NGS, Sanger sequencing is still used as the gold standard technique for variant confirmation and segregation analysis in trios (patient, mother, and father), which is necessary to define whether two variants are found in cis or trans (variants present in the same or in opposite DNA strands, respectively) when an autosomal recessive disease is suspected. Additionally, the segregation of a variant in large families or groups of patients sharing a certain phenotype and its absence in non-affected individuals is a very important piece of evidence supporting its pathogenic role.

WES and WGS may lead to the identification of both genes of unknown significance (GUS) and variants of unknown significance (VUS) [27]. The first term refers to variants identified in genes not previously associated with the disease, while the second term refers to a variant whose impact on protein function is not yet known. Assigning a pathogenic role to those variants is not possible, even if the type of variant is clearly LOF, as we cannot be certain if LOF is a mechanism of disease for that specific gene. In those cases, a useful strategy is to share observations regarding specific rare variants with the clinical genetics community to identify additional patients across the world with similar phenotypes. For that purpose, tools such as Matchmaker Exchange from the Global Alliance for Genomics and Health (GA4GH) [28] or MyGene2 (https://www.mygene2.org, accessed on 9 July 2021) have been developed.

Genetic variants in coding regions may affect gene and/or protein function by several different mechanisms. A priori, frameshift and nonsense variants are usually considered as deleterious and probably LOF because of degradation of mRNA by NMD or protein truncation but it is well known that some variant alleles may escape NMD and function as benign or GOF alleles [9,29]. Missense SNVs are the most common type of variant and constitute the vast majority of VUS found by NGS. The effects of synonymous SNVs, which do not change the amino acid residue, on the molecular functionality of the corresponding genes and proteins are often assumed to be minimal. However, it has been well established that silent variants may disrupt transcription, alter mRNA stability, and directly affect splicing by activating cryptic splice sites or disrupting canonical ones, or by affecting exonic splicing enhancers (ESE) or exonic splicing silencers (ESS), thus modulating gene expression. Interestingly, missense variants might also affect splicing. When performing WGS, the effects of gene variants found in intronic, or regulatory regions are even more difficult to predict. The study at the mRNA level results in a very interesting approach to demonstrate the consequences of this type of gene variant [30]. However, this is only possible when the gene of interest is expressed in an easily accessible tissue, and it also depends on the temporal expression pattern of the particular gene being analyzed.

The identification by NGS of such a huge number of unreported variants together with difficulties associated with defining their potential LOF or GOF effect has enormously increased the number of variants that need to be evaluated by functional assays. This has prompted the development of an ever-growing number of bioinformatic tools to predict their potential deleterious effect on gene and protein function. Many bioinformatic prediction methods are widely used not only to provide additional evidence for variant prioritization but also to help to select which variants might be the best candidates for in vitro and/or in vivo studies, which are quite resource- and time-consuming. Briefly, bioinformatic tools aim to evaluate three main aspects of variants: evolutionary conservation, the effect of amino acid changes on protein structure and function (only for missense variants) and their effect on splicing. Additionally, there are predictors able to evaluate the possibility of NMD escape for frameshift and truncating variants. However, to properly apply these tools, it is important to understand how these predictions work, on which data they are based, what the results mean, and how reliable they are [31]. As expected, given the different nature of the algorithms underlying each predictor used to infer variant pathogenicity, concordance is far from being perfect among them, especially for benign variants, thus hindering the use of in silico evidence. Frequently used population, disease, sequence, and expression databases and Web based useful resources are listed in Table S2. ACMG recommendations suggest using several in silico programs, based on different algorithms, to evaluate each variant. If most of them agree on the prediction, this evidence can be counted as supporting but if they disagree, this evidence should not be considered to classify the variant [8]. A better alternative seems to be the use of more recently developed metapredictors, such as REVEL, VEST3, MetaLR, MetaSVM, M-CAP, CADD, etc., which achieve higher predictive power by combining multiple individual predictors. However, the implementation of these newer tools by clinical genetic laboratories is not yet as spread as previous ones.

In recent years, many bioinformatic tools for CNV detection and calling from NGS data have been developed [31,32,33,34]. Most of them show better performance when working with WGS or WES data and for large CNV detection. Thus, the detection of small CNVs affecting only one or a few exons from NGS gene panels data, which are more widely used in routine genetic testing, is still challenging. Methods for CNV detection using NGS data can be based on different principles. Some of the most used algorithms, like DECoN and Exome Depth, rely on the comparison of the read depth in a sample with a group of controls assuming that the number of reads mapped to a certain region is proportional to the number of copies, comparing the depth of coverage in a sample with a group of controls. Other methods depend on paired-end mapping of reads, split-reads or assembly [35].

Thus, molecular diagnosis need to be based on accurace and consistent classification of sequence variants [36].

Variant classification has evolved over time since the first report of the ACMG in 2000 [37]. By that time, the expert working group recommendations aimed to provide a framework for the interpretation and clinical reporting of sequence variants in one of five categories: (i) Sequence variation is previously reported and is a recognized cause of the disorder, (ii) Sequence variation is previously unreported and is of the type, which is expected to cause the disorder (nonsense, frameshift, splicing), (iii) Sequence variation is previously unreported and is of the type, which may or may not be causative of the disorder, (iv) Sequence variation is previously unreported and is probably not causative of disease (silent, not affecting splicing), (v) Sequence variation is previously reported and is a recognized neutral variant. In 2008, an updated version of ACMG recommendations was published, adding a sixth category: (vi) sequence variation is previously not known or expected to be causative of disease, but is found to be associated with a clinical presentation [38]. Even though efforts were made to guide variant interpretation and reporting, variant classification is not a simple or straightforward process and considerable discrepancy exists among different laboratories. By 2015, a working group formed by experts from the ACMG and the College of American Pathologists (CAP) published a new consensus report standardizing the process of variant classification based on expert opinion, in silico predictive data and empirical data such as population frequency, functional studies and segregation data. The specific standard terminology proposed included the following five categories to describe variants identified in genes that cause Mendelian disorders: “pathogenic”, “likely pathogenic,” “uncertain significance,” “likely benign,” and “benign” [8]. Since its publication in 2015, ACMG/CAP recommendations for variant classification have been widely adopted by the scientific and the clinical community, but still, many discrepancies remain. Variants in the uncertain significance category (VUS) are the most challenging ones because there is not enough evidence available to classify them as (likely) benign nor as (likely) pathogenic. This group of variants requires a close follow-up over time to check for evidence updates in literature reports or disease-specific databases, which allow one to define if they can be considered as disease-causing or not.

Similar criteria and difficulties apply to CNV variants classification. Recently, the ACMG and the Clinical Genome Resource (ClinGen) published a joint consensus recommendation for the interpretation and reporting of CNVs [39], where they have updated the previous 3-tiered system of clinical significance [40] to a system using the same five categories (pathogenic, likely pathogenic, uncertain significance, likely benign and benign) applied to small insertions, deletions and single nucleotide variants (SNVs).

**Figure 2:**
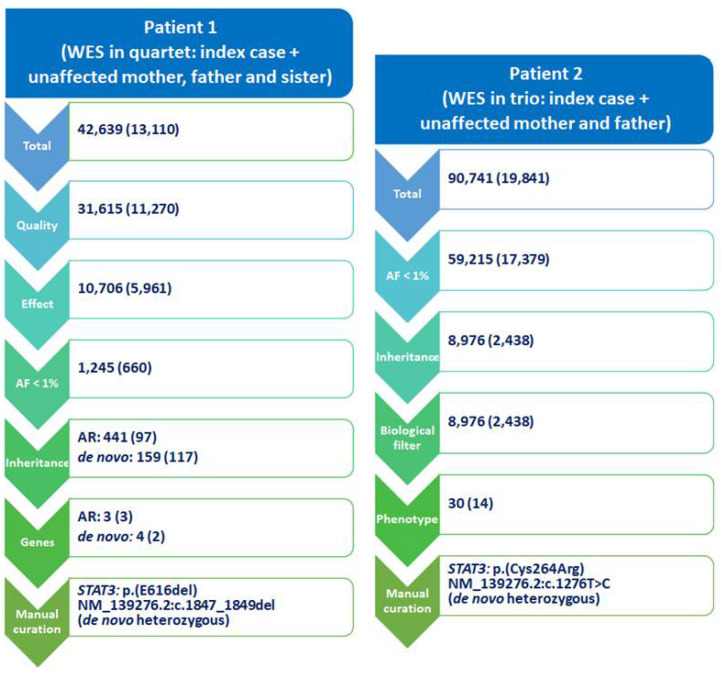
Different filtering strategies applied to WES data analysis. Number of variants obtained in each filtering step is indicated, with the number of genes in parentheses. The sequential filtering strategy which led to the prioritization of probably pathogenic candidate variants, to explain the patients’ phenotype of severe short stature associated with immune dysregulation, was different for each case. For patient 1, variants considered fulfilled the following criteria: adequate quality, an effect on the protein (either located in coding region: nonsense, missense, in-frame deletions or insertions, and frameshift variants), allelic frequency (AF) < 1% in the general population (according to ExAC), consistent with the presumed inheritance pattern (either autosomal recessive (AR) with two variants in different alleles of the same gene or de novo variant were suspected, since her parents and sister were unaffected), and variants included in a 250-candidate gene list. For patient 2, filtering criteria for variants present in the trio included: AF < 1%, AR or de novo inheritance pattern, a biological filter (genes related to lymphocyte activation), and phenotype (eczema, colitis, autoimmune thyroiditis, autoinflammatory disorders). For both cases, a final step of manual curation of several variants was required to select the prioritized *STAT3* variant.

In our experience, bioinformatic analysis applying different filtering strategies (Figure 2) allowed us to prioritize two *STAT3* variants (NM_139276.2: c.1276T>C, p.C426R and NM_139276.2: c.1847_1849delAAG, p.E616del) in two patients with short stature and immune dysregulation studied by WES in a quartet (index case and unaffected mother, father and sister) or trio design in 2018 [41]. Multiple sequence alignment from different species showed that both residues C426 and E616 are highly conserved within the DNA binding and the SH2 domains, respectively, suggesting an important role of that position for proper protein function. Variant p.C426R was predicted as deleterious by three out of five bioinformatic predictors used, but p.E616del could only be analyzed with one of the five tools employed (Mutation Taster). According to the evidence available at that moment, and following the ACMG guidelines, both variants were classified as likely pathogenic. Molecular modelling further supported their pathogenic role and in vitro functional studies later confirmed GOF effect for both variants (refer to Section 4. In vitro functional studies, Intracellular Transcription Factors for further details).

## 3. In Silico Modeling

A majority of the nonsynonymous SNVs associated with human disorders are caused by an alteration in structural stability [42,43]. Disease-causing missense variants tend to disturb hydrogen bonding networks and disulfide and salt bridges, thus altering the native state of the protein. Proteins go through a series of folding to achieve their lowest free energy values. Thus, disease-causing variants might inhibit this process yielding higher free energy proteins. Misfolded, non-native proteins can be processed by chaperone-mediated autophagy and/or degraded in the cytosol through the ubiquitin–proteasome pathway [44]. In vitro experiments visualize these proteins as the absence of bands in SDS-PAGE gels and can be similar to the absence of bands of null protein genetic variants [45,46]. The stability of a protein may be directly related to its functional activity and incorrect folding and decreased stability can be the major consequences of pathogenic missense variants [47,48].

Protein–protein-interacting interfaces are usually referred to as binding hot spots of proteins. These regions are charged, structurally conserved and highly polar and are surrounded by hydrophobic residues, which are the residues that are mostly involved in the binding [49]. Variants may affect the electrostatic nature of protein surfaces and introduce changes in stability or folding, altering binding partner specificity and affinity, changing protein function [49].

The term in silico goes back to the 1970s and is related to the computer component silicon. In silico methods are based on computational approaches for the prediction of effects prior to the development of laboratory methods [50]. Presently, the most rapid and inexpensive way to predict whether a missense variant will potentially cause disease is by performing computational analysis. Ideally, any method used to assess the pathogenicity of genomic variants should possess the ability to look beyond genomic annotation to a functional context, at an atomic resolution. Besides sequence conservation, various other sequence and structural features are used, including changes in physicochemical properties between wildtype (WT) and substituted amino acid, structural features (mostly solvent accessibility), site mutability in DNA, and sequence context of the site. The most obvious advantages of computational approaches include the potential to test hypotheses in silico that would be difficult, costly, or intractable in the lab [51]. In fact, in silico prediction of the amino acid substitution impact at the protein level may, sometimes, be considered as an alternative to in vitro expression or as a pre-study indicator of the need for research at the functional level.

The impact of genetic variants on protein structures can be analyzed using three-dimensional (3D) structures, molecular modeling, and molecular dynamic simulations. Molecular modeling allows us to visualize the manner in which proteins are folded to create a functional structure and to accurately simulate how this is disrupted by mutational events. Nonetheless, for making a reliable molecular model, it is essential to know the protein biology, function and interaction with other cellular components. In addition, the generation of a model usually depends on the existence of the experimentally determined macromolecular structure or, at least, a partial homologous structure using either X-ray crystallography, NMR spectroscopy or electron microscopy. Since such techniques are very time-consuming and expensive, modeling through bioinformatic programs has managed to predict the atomic structure of several proteins from their amino acid sequence, by comparison with known protein structures, where the processes are faster and more economical [52,53]. If the protein studied presents a homolog of known structure, the analysis is easier and the generated model is of higher resolution, but if the homologs do not exist or are not identified, the modeling is constructed from scratch [54]. Experimental exploration of different positions in a protein structure with various residue types is a time-consuming and expensive process. Such an exploration is generally facilitated by 3D modeling of side-chain mutations [55,56,57,58]. Seemingly insignificant change of a sidechain may lead to a significant change or loss of protein function [59]. Homology modeling methods allow for prioritizing the most interesting and likely pathogenic cases for further experimental analysis when the number of variants identified is high. Finally, predicting the phenotypic effect of SNVs using in silico methods may provide a greater understanding of genetic differences in susceptibility to disease.

**Figure 3:**
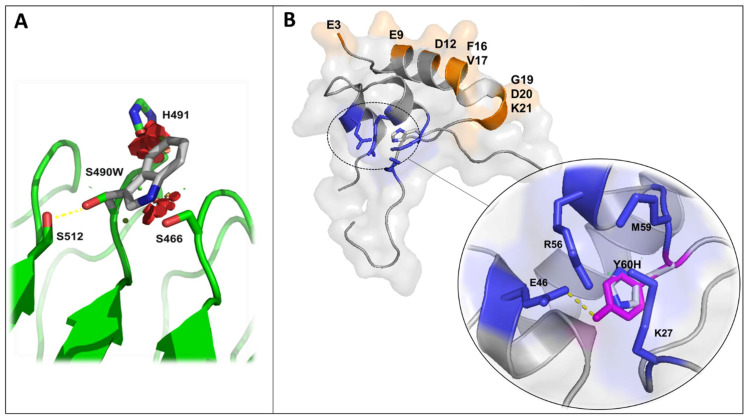
Molecular models of ALS and IGF1 variants. In silico substitutions were performed using PyMol Mutagenesis Wizard and side-chain rotamers were chosen to avoid steric clashes with the rest of the residues. Selected residues are represented in stick and labeled. (**A**) p.S490W-ALS variant. The tryptophan side chain (gray) is displayed as an overlay of the WT side chain (green). Loss of hydrogen bond with S512 and steric clashes (depicted as red disks) are predicted after S490W mutation, possibly leading to overall destabilizing effects. (**B**) Cartoon representation of p.Y108H-IGF1 variant structure (equivalent to p.Y60H in the mature peptide). Y60 (showed in magenta) is an identified residue that interacts with IGF1R. The hydroxyl group of Y60 forms a hydrogen bond (dotted yellow lines) with E46 that is lost after tyrosine for histidine substitution (gray residue). Key residues involved in IGFBP-3 interaction (showed in orange) are located on the surface of IGF1 far from the p.Y60H change.

In our own experience, the association between the in silico approach and in vitro experimentation has been rather evidenced by several previous studies. A widely used software for molecular visualization and in silico modeling is PyMOL (PyMOL Molecular Graphics System, Version 1.8.4.0, Schrödinger, LLC, http://www.pymol.org/, accessed on 15 July 2021). The PyMOL program was used for in silico structural analysis of eleven *IGFALS* variants identified in short-statured children [60], two activating *STAT3* variants in two patients with short stature and immune dysregulation [41], one homozygous variant in *IGF1* in a patient with short stature and bilateral sensorineural deafness [61], and a heterozygous *STAT5B* variant in a patient with short stature [62]. Of these, 3D structural analysis of the substitution context has been especially helpful for those variants where bioinformatic prediction software yields inconsistent results, with classifications ranging from pathogenic to benign. For example, p.S490W-ALS was predicted as a pathogenic variant by two out of four pathogenicity prediction tools [60]. However, in silico structural analysis showed that a serine to tryptophan substitution would have an overall structural destabilizing effect due to the loss of the hydrogen bond with Ser512 and the introduction of the tryptophan large bulky aromatic side chain that clashes with surrounding residues (Figure 3A). In vitro functional experiments supported the impact of this mutation on protein folding, since this variant was absent in lysates and cell media, suggesting it is intracellularly degraded as a consequence of an aberrant non-native conformation [60].

For variants homogeneously classified as pathogenic using multiple bioinformatics tools, structural analysis helped us to gain biological insights into the effect of these pathogenic variants. For instance, the IGF1 variant p.Y108H was consistently classified as pathogenic using multiple bioinformatics tools [61]. The structural analysis of this substitution predicted conformational changes in the receptor-binding domain, impairing the type 1 IGF Receptor (IGF1R) activation, but not IGFBP-3 binding (Figure 3B). In line with this, in vitro studies using serum from the patient showed decreased ability of IGF1 p.Y108H to activate IGF1R compared to age-matched controls. However, when IGFBPs were removed from the patient’s serum by acid chromatography, the phosphorylated band of IGF1R was stronger than in the presence of IGFBPs, suggesting that in the patient´s serum IGF1 p.Y108H was mainly bound to its binding proteins, as predicted by the molecular model [61]. Therefore, while variant pathogenicity bioinformatic prediction tools may classify variants with reasonable accuracy, the evaluation of structural effects of mutational changes could help to understand how variants impact protein function in a clinically relevant context.

## 4. In Vitro Functional Studies

**Figure 4:**
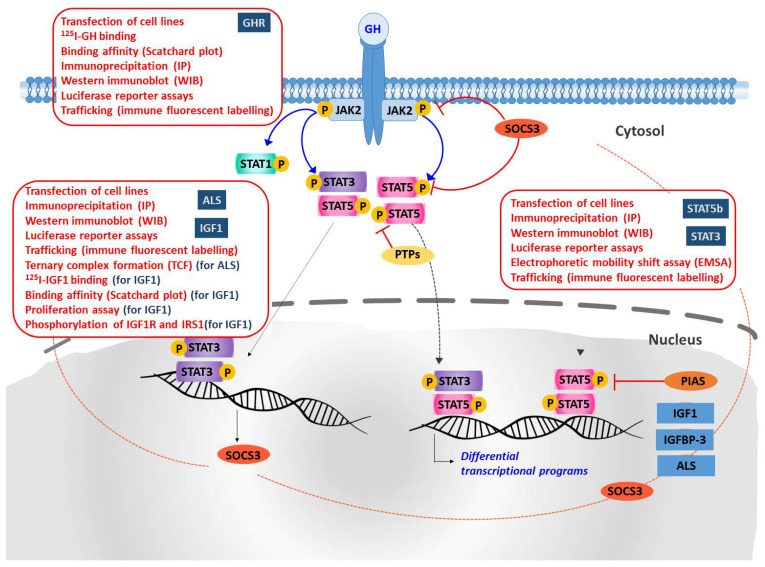
Schematic representation of GH action and the methodological tools applied to the characterization of genetic variants identified in different molecular players. Upon GH binding to dimerized cell-surface GH receptor (GHR), JAK2 is activated and phosphorylates specific tyrosine residues in the intracellular domain of the receptor. STAT proteins bind to these phosphotyrosine motifs through their SH2 domain, become phosphorylated (STAT-P), form homo and heterodimers, and translocate to the nucleus. In the nucleus, STATs dimers bind to specific promoter elements and regulate gene expression. Expression of insulin-like growth factor-1 (IGF1), insulin-like growth factor binding protein-3 (IGFBP-3), and acid-labile subunit (ALS) are upregulated by STAT5 homodimers. Negative regulation of STATs activation is mediated by SOCS (suppressors of cytokine signaling), PIAS (protein inhibitors of activated STATs) and PTPs (protein tyrosine phosphatases).

Determining the function of a given protein requires investigating several biological aspects. Although immortalized cell lines offer a fast and inexpensive method for studying potentially pathogenic variants identified in a given genetic disease, they can sometimes not be representative of what actually occurs in vivo. The use of human primary cell lines is a better choice in order to mimic the physiological conditions of a given tissue. These cell lines usually retain all the morphological and functional characteristics of the tissue of origin. In vitro techniques following transfection of cell cultures using WT and mutant cDNA include immunoprecipitation (IP) and western immunoblot (WIB) to detect correct expression of these and other downstream mediators, binding assays, luciferase (Luc) reporter assays using reporter vectors under hormone stimulation and immunofluorescence (IF) assays to study intracellular trafficking of mutant proteins (Figure 4).

Regardless of the nature of the gene to be studied, the first step when developing an in vitro functional assay is always to have the human cDNA of the gene under study cloned into an expression vector such as pcDNA3.1, which has a multiple cloning site, a eukaryotic promoter such as CMV, a polyadenylation sequence, and an antibiotic resistance gene for bacterial selection. The next step is to introduce the desired mutation into the cDNA within the expression vector using site-directed mutagenesis. Selecting the appropriate cell line is also challenging and, usually, it is better if the cell line does not express our gene of interest so that the expression of the transfected gene can be easily controlled. Reconstitution studies rely on the transfection of cell lines using either the calcium-phosphate method or lipidic molecules such as lipofectamine reagent (Invitrogen, Carlsbad, CA, USA). Choosing the appropriate downstream methods depends on the nature of the protein being studied: membrane-bound receptors, intracellular transcription factors, soluble secreted proteins, or soluble secreted enzymes.

### 4.1. Membrane-Bound Receptors

If the gene of interest encodes a membrane-bound receptor, in vitro reconstitution studies can be performed after co-transfecting with both WT and mutant vectors followed by IP and WIB using specific antibodies in order to determine the correct expression of the mutant receptor on the cell surface. In those cases, in which the receptor is internalized, it would be also possible to follow up the trafficking of the receptor by using immunofluorometric (IF) assays. For example, mutations in the GH receptor (*GHR*) gene, a membrane-bound receptor, lead to complete GH insensitivity (GHI) which is inherited as an autosomal recessive condition. Patients with mutations in this gene present Laron Syndrome (LS) characterized by extreme short stature with high levels of GH [63]. The first molecular characterization of two patients with Laron-type dwarfism showed that they have a deletion of a large portion of the extracellular domain of the receptor gene. Interestingly, this deletion includes nonconsecutive exons, suggesting that an unusual rearrangement may have occurred [64]. It is also worthy to emphasize that even synonymous genetic variants may sometimes have a profound impact on the coding protein. An example of this was the characterization of a large pedigree of patients with LS in Southern Ecuador [65]. Sequencing of the *GHR* gene revealed the substitution of guanine for adenine in the third position of codon 180 that did not change the glutamic acid amino acid encoded. Sequencing of the exon 6–exon 7 splice junction from RNA-polymerase chain reaction, which amplified cellular RNA of an affected individual revealed that the substitution activates a 5′ splice site 24 nucleotides upstream from the normal exon 6–intron 6 boundary. This results in a splicing mutation with the consequent loss of eight amino acid residues in the extracellular domain of the GHR protein that abolished binding affinity for GH, rendering the GHR completely inactive (p.E180splice) [66]. The p.E180splice is the most frequent GHR mutation and has also been reported in patients from Israel, Brazil, Chile, and the United States. Intragenic polymorphisms and Y-chromosome single-tandem repeat polymorphism (STRs) confirm a common ancestor, which probably originated in the Iberic peninsula that later spread around the world with the exodus of the Sephardic population [67]. In addition, partial GHI is observed in some patients with heterozygous variants in this gene with a dominant negative effect [68,69,70]. The first in vitro functional characterization of a dominant negative mutation in the *GHR* gene (c.876G>C affecting the 3′ splice acceptor site preceding exon 9) was developed by Ayling et al. [68]. The authors were able to demonstrate using IP that WT and mutant GHR form heterodimers upon ligand binding. In addition, binding assays can be performed to determine if the receptor is capable of binding its ligand. Unfortunately, this type of assay does not evidence the potential activation of the receptor upon ligand binding. Binding assays are performed using ^125^I-hGH and afterward bound and free hGH are separated by centrifugations and specifically bound ^125^I-hGH in the pellet is determined using a gamma-counter followed by Scatchard analysis. Wojcik et al. identified four missense variants (p.D152H, p.I153T, p.Q154P, and p.V155G) located in the extracellular domain of the GHR in four different patients with LS, three of which were novel. Transfection studies in HEK293 cells showed that cells expressing p.I153T, p.V155G, and p.Q154P mutants had lower hormone-binding affinity and intracellular trafficking defects while p.D152H affected receptor expression, dimerization, and signaling [71]. Finally, Luc reporter assays to analyze GHR downstream activation of STAT5b, the major mediator of the GHR signaling pathway, generally use reporter vectors with STAT5b binding sites. These assays allow for the analysis of both homozygous (WT only and mutant only) and heterozygous (WT and mutant) alleles mimicking a patient’s genotype. The possibility of co-transfecting both WT and mutant alleles allows the study of dominant negative mutations. For example, Ayling et al. performed co-transfection studies of both mutant and WT receptors together with a reporter gene containing STAT5b binding sites and showed that the mutant protein was unable to activate STAT5b. In addition, the mutant protein exerted a dominant negative effect on the WT protein [68]. Fang et al. reported two female patients with GHI who were compound heterozygous for two genetic variants in the GHR gene (p.C94S and p.H150Q) [72]. The authors performed all of the above-mentioned techniques. WIB and GH binding assays were performed in HEK293 cells after transfection of mutant and WT cDNAs. WIB showed that p.C94S did not show the higher molecular weight of the normally glycosylated form of GHR, while binding assays confirmed that the variant had a lower specific affinity for GH (6% of WT binding affinity) in its homozygous state, but its binding affinity was similar to WT when in a heterozygous state with WT GHR. Meanwhile, variant p.H150Q had a similar binding affinity as WT in its homozygous state but when in heterozygosis with p.C94S it had 50% of binding affinity of that observed with WT GHR. Interestingly, both mutants in their homozygous state showed a reduction in STAT5b activation upon induction with GH. While STAT5b activation was normal when each variant was co-expressed in heterozygosis with WT, the co-expression of p.C94S/p.H150Q led to significantly reduced levels of phospho-STAT5b. Primary dermal fibroblasts were analyzed in one of the patients showing similar results of impaired activation of STAT5b. In addition, Luc reporter assays determined that cells transfected with p.C94S in homozygosis had an absence of Luc activity upon GH stimulation, while p.H150Q transfected cells did not impact Luc activity but p.C94S/p.H150Q double heterozygotes showed markedly reduced Luc activity [72]. Finally, another example of the use of the downstream techniques mentioned above to functionally characterize mutations in the *GHR* gene was shown by Derr et al. [73]. Aisenberg et al. had previously reported a patient with GHI, who was compound heterozygous for c.686G>A/c.899dup (p.R229H/p.V301Sfs*7) (this last mutation results in a frameshift and a premature stop codon) variants in the GHR [74] and Derr et al. performed in vitro functional studies to characterize their pathogenicity in order to infer causality [73]. The authors demonstrated that the variant p.R229H was not only correctly expressed but also was capable of activating STAT5b and Luc activity similar to WT thus behaving like a benign variant. On the other hand, variant c.899dupC displayed no GH-induced activation suggesting it may act in a dominant negative manner. Further evidence of this dominant-negative effect was obtained by the co-expression of p.V301Sfs*7/p.R229H or p.V301Sfs*7/WT cDNA that resulted in a significant reduction in GH-induced STAT5b activation and transcriptional activity using WIB and Luc reporter assays [73].

### 4.2. Intracellular Transcription Factors

When the gene of interest encodes an intracellular transcription factor, downstream methods used include IP and WIB to determine if mutants are expressed at the same level as WT proteins. The choice of antibody used for these procedures is important since it must detect both WT and mutant proteins. Luc reporter assays using reporter vectors with specific transcription factor binding sites are useful to study the transcription factor’s capability to bind and activate transcription. Finally, IF assays to study the intracellular trafficking of the protein of interest turned out to be extremely informative. For this, both WT and mutant cDNAs must be differentially fluorescently tagged in order to detect the intracellular localization of the encoding proteins, either nucleus or cytoplasm. Fluorescently labeled secondary antibodies are used to treat fixed cells and co-localization studies can be performed by mixing WT and mutant cDNA in a 1:1 ratio in order to study heterozygotes. The most commonly studied transcription factor belonging to the GH-IGF1 pathway is STAT5b. The *STAT5B* gene encodes signal transducer and activator of transcription 5 and is the key mediator of GH growth-promoting actions. Pathogenic genetic variants in this gene are associated with severe short stature, GHI and a moderate to severe immune dysfunction and are inherited in an autosomal recessive mode [75]. Scaglia et al. identified a novel *STAT5B* homozygous variant in a female patient with GHI (p.F646S located in the SH2 domain) and analyzed its functionality using reconstitution studies together with a previously reported homozygous mutation (p.A630P also located in the SH2 domain) [76]. Reconstitution studies were performed using expression vectors with both variants and WT STAT5b. IP followed by WIB revealed that both variants were expressed at lower levels compared to WT STAT5b. Upon GH stimulation this transcription factor becomes phosphorylated, dimerizes and translocates to the nucleus. By using anti-P-STAT5b antibodies WIB enables the detection of STAT5b phosphorylation. In this work, phosphorylation of both variants was less robust compared to WT. In vitro Luc reporter assays were performed using a GH-induced reporter vector and both variants p.F646S and p.A630P were unable to drive luciferase activity throughout the GH response element (GHRE) compared to WT. One copy of the WT *STAT5B* allele seems to be sufficient for normal height since heterozygous relatives of affected patients are of normal height and without immunological complications [77]. Klammt et al. reported the first heterozygous *STAT5B* variants with dominant-negative effect identified by WES in three patients from three unrelated families (p.Q177P, p.A478V, and p.Q474R) [78]. The patients had short stature and mild immunological defects. These variants were functionally studied in vitro in HEK293 cells, and all three variants showed an expression level comparable to WT STAT5B. Co-IP showed homo- and hetero-dimerization capabilities for each variant with WT STAT5B. In contrast to the other two variants, p.Q177P was unable to translocate to the nucleus as shown by IF studies thus retaining the WT allele in the cytoplasm when dimerized. Meanwhile, p.Q474R and p.A478V were unable to bind to target DNA, which was detected using electrophoretic mobility shift assay (EMSA) analysis using a DNA-probe (GHRE) under GH-stimulation. Finally, results of Luc reporter assays evidenced that neither variant could drive expression of the Luc reporter by themselves and when co-expressed with WT STAT5b, the transcriptional activity of WT STAT5b was significantly lower compared to WT STAT5b alone. One year later, Ramirez et al. reported a patient with short stature and a mild immunological phenotype due to two heterozygous variants in the GH-IGF1 signaling pathway, a novel missense variant in the *STAT5B* gene (p.K632N) and a previously described missense variant in the *IGFALS* gene (p.R548W) identified by WES [62]. HEK293-T cells were used to transiently express the mutant and WT STAT5b expression vectors. WIB showed comparable levels of mutant and WT protein. However, GH-induced phosphorylation of the p.K632N variant was not detected. In addition, cells co-expressing both WT and p.K632N proteins showed reduced levels of STAT5b phosphorylation. Luc reporter assay was used to investigate the transcriptional activity of the variant. Under unstimulated conditions, the p.K632N variant resulted in a significant decrease in reporter activity compared to WT-STAT5b. When both were co-transfected in equimolar amounts, there was also a decrease in transcriptional activity compared to WT alone. Surprisingly, under GH stimulation, even though variant p.K632N was unable to drive Luc reporter activity, co-transfection of both p.K632N and WT-STAT5b showed a transcriptional activity similar to WT alone. Immunofluorescence studies demonstrated that after GH-stimulation, p.K632N-STAT5b remained in the cytoplasm while WT-STAT5b was translocated to the nucleus. Interestingly, the patients presented a positive response to recombinant human-GH (rhGH) indicating that the partial GHI can be overcome by pharmacological doses of rhGH.

Another important transcription factor involved in the GH-IGF1 pathway is STAT3. The *STAT3* gene encodes signal transducer and activator of transcription 3. Heterozygous GOF or activating variants in the *STAT3* gene have been reported in patients with immune dysregulation and growth failure, normal GH levels and low IGF1 levels [41,79,80,81]. Flanagan et al. [79] sequenced the *STAT3* gene in 24 patients with autoimmune disorders of unknown cause and in 39 individuals with isolated permanent diabetes and identified four different heterozygous missense variants in five individuals (p.K392R, p.N646K, p.K658N, and p.T716M [80]. They all were de novo mutations. The identified variants were evaluated using a STAT3 responsive dual-Luc reporter assay alongside two previously reported dominant-negative hyper-IgE syndrome inactivating *STAT3* gene mutations and WT STAT3. Results showed that under basal non-stimulated conditions all four identified mutants had increased Luc activity compared to WT STAT3 and to the hyper-IgE inactivating mutations. After stimulation with IL-6, three of the four variants (p.K392R, p.K658N, and p.T716M) showed an increase in Luc reporter activity, but neither p.N646K nor WT STAT3 showed an increase in the Luc reporter activity beyond basal levels. Just one year later, Milner et al. [81] identified 10 genetic variants (some of them novel and others previously described) in 13 patients with early-onset autoimmunity and lymphoproliferative disease from 10 families: p.G421R, p.T663I, p.R152W, p.V353F, p.Q344H, p.E415K, p.T716M, p.N420K, p.A703T, and p.T716M) [80]. Functional in vitro studies were performed in STAT3 deficient cells (A4 cells). WIB showed that all mutant proteins were expressed and detected except for p.A703T mutation, which was only detected using a different anti-STAT3 antibody. Phosphorylation of STAT3 mutants was not significantly increased under basal conditions, and it was normal under IL-6 stimulation. A STAT3 dual-Luc reporter assay showed that all mutants except for p.V353F had significantly increased activity at basal levels compared to WT STAT3. Nevertheless, all mutants showed increased Luc activity after stimulation with either IL-6 or IFN-alpha. A major downstream target of STAT3 is SOCS3, which in turn is an inhibitor of STAT3. Four patients´ immortalized fibroblasts were studied under basal levels and after stimulation with IL-21 and QRT-PCR was performed to measure SOCS3 levels. Transcript levels of SOCS3 were elevated both at baseline and after stimulation even for mutant p.V353F, which did not show baseline activity in Luc reporter assays highlighting differences between reconstitutions in vitro studies and endogenous expression from a patient-derived cell line.

More recently, Gutiérrez et al. [41] identified two novel *STAT3* genetic variants (p.E616del and p.C426R) in two unrelated patients with IGF1 deficiency and immune dysregulation using WES [59]. Functional analysis showed that both were GOF mutations. WIB was performed after transfection of HEK293-T cells expressing GHR with mutant and WT STAT3 constructs. STAT3 was not phosphorylated under basal conditions in either mutant as well as WT STAT3. On the other hand, both mutants and WT STAT3 were phosphorylated upon stimulation with IL-6 and GH. Both mutants were still phosphorylated after 120 min of GH treatment, while at that time phosphorylation of mutant-STAT3 was markedly diminished in WT STAT3, showing different dephosphorylation kinetics under GH or IL-6 treatments. While p.C426R showed delayed dephosphorylation only under GH treatment, p.E616del showed delayed dephosphorylation only under IL-6 stimulation.

### 4.3. Soluble Secreted Proteins

For the study of soluble secreted proteins, once the cell line is transfected with WT and mutant cDNA vectors, it is important to separate and study both lysate and conditioned medium through WIB. This allows the differentiation between mutants with defects in their expression and mutants with defects in their secretion. When studying secreted binding proteins, binding assays can be performed using the purified conditioned medium following reconstitution studies. The Acid-labile Subunit (ALS) is a protein belonging to the Leucine-Rich Repeats (LRR) family of proteins and its role is to stabilize IGF1 and prolong its half-life in the vascular compartment. It does this by forming ternary complexes with either IGFBP-3 or -5 and IGF1 [82].

Homozygous and compound heterozygous genetic variants in the *IGFALS* gene results in complete ALS deficiency, characterized by severe primary IGF1 and IGFBP-3 deficiencies and complete GHI but mild growth retardation [83]. A recessive pattern of inheritance has been described in this condition [84,85]. Heterozygous *IGFALS* gene mutations have also been described in children showing a phenotype previously labeled as idiopathic short stature (ISS) [86].

Functional in vitro characterization of several *IGFALS* variants has shown that pathogenic variants result in the absence of ALS synthesis or intracellular retention of the mutant protein. Firth et al. studied a missense variant (p.D440N) using recombinant adenoviruses expressing WT or p.D440N-ALS [87]. Even though the mutant protein was detected intracellularly by WIB, it was absent in the conditioned media suggesting a secretion defect. Intracellular p.D440N-ALS was unable to form ternary complexes with ^125^I-IGF1-IGFBP-3. Functional in vitro studies of eleven variants identified in children with ALS deficiency (ALS-D) or with ISS were performed by transient transfection of CHO cells [60]. WIB showed that ALS variants p.P287L, p.A330N, p.A475V, and p.R548W were present both in cell lysates and cell media with a similar molecular weight as WT ALS. However, variants p.E35Kfs*87, p.E35Gfs*17, p.N276S, p.L409F, p.S490W, and p.C540R were undetectable either in cell lysates or cell media. Finally, p.L213F-ALS was present in cell lysates but not in cell media indicating a secretion defect. In vitro ternary complex formation studies of secreted variants (p.P287L, p.A330N, p.A475V, and p.R548W) indicated that all variants retained their ability to form ternary complexes in vitro. For in vitro ternary complex formation assays, conditioned media from cells transfected with WT or mutant ALS is incubated with rhIGFBP-3 and ^125^I-IGF1 followed by size exclusion chromatography and ternary complex formation is evaluated as the ratio between the area corresponding to the ternary complex and total area under the elution curve.

When studying soluble secreted proteins such as hormones, which act as ligands for a particular receptor, binding affinity can be determined using a radiolabeled ligand binding assay. For this, lysed cells overexpressing the receptor, or a soluble form of the receptor are used to determine the competition between the radiolabeled ligand versus WT or mutant ligand from reconstitution assays from cells transfected with each ligand. Binding kinetics are determined by Scatchard analysis. An example of such a ligand in the GH-IGF1 pathway is IGF1 itself. The first patient with total IGF1 deficiency was a male, who had a homozygous deletion of exons 4 and 5 of the IGF1 gene [88]. Complete IGF1 deficiency is characterized by severe pre- and postnatal growth retardation, microcephaly, neurosensorial deafness and mental retardation. Walenkamp et al. described a 55-year-old male from consanguineous parents, who presented severe intrauterine and postnatal growth retardation, microcephaly, and sensorineural deafness, identifying a missense variant (p.V44M) in the *IGF1* gene [89]. In vitro binding showed that p.V44M-IGF1 had a 90-fold lower affinity for the IGF1R compared with WT IGF1. The p.V44M-IGF1 was measurable in both europium immunometric and ^125^I-labeled IGF1 competition receptor binding assays. In addition to binding assays, proliferation assays can also be performed for IGF1. Such assays can be performed by measuring ^3^H-thymidine incorporation in a particular cell line supplemented with serum samples from the patient or normal controls or by directly counting 0.5% trypan blue-stained cells before and after supplementation with patient and control serum samples. Finally, phosphorylation of the IGF1R and the insulin receptor substrate 1 (IRS-1) can also be performed to study the signal transduction ability of IGF1 in vitro. For this, proteins from cell cultures supplemented with serum from patients or controls are separated by SDS-PAGE and immunoblotted with anti-phosphotyrosine antibodies corresponding to either IGF1R or IRS-1. In 2009, Netchine et al. studied a boy with intrauterine growth restriction and postnatal growth failure, microcephaly and normal hearing who was homozygous for a missense variant (p.R36Q) [90]. He had reduced levels of IGF1, with IGFBP-3 and ALS in the upper normal range. Cell proliferation assays were performed using serum-starved BP-A31 cells by monitoring the incorporation of ^3^H-thymidine after stimulation with varying concentrations of either WT or mutated IGF1 peptides. Two times higher mutant IGF1 concentrations were necessary to produce proliferation levels compared to WT IGF1. Binding assays were performed using a soluble form of IGF1R showing that the p.R36Q-IGF1 had only a moderately lower affinity for the receptor than WT. Finally, the authors analyzed the effect of p.R36Q mutation on IGF1 signal transduction by monitoring the phosphorylation of IGF1R and IRS-1 in MCF-7 cells and evidenced that the mutant IGF1 was less effective than WT IGF1 suggesting that signal transduction is impaired. More recently, Keselman et al. reported a boy born from consanguineous parents with intrauterine growth restriction and severe postnatal growth with a *IGF1* homozygous missense variant p.Y108H [61]. Using HEK293 cells, the patient’s serum was studied to evaluate its ability to induce IGF1R phosphorylation leading to a weaker stimulation by mutant IGF1. In addition, cell proliferation was also evaluated in response to the patient’s serum and results showed that while control samples were able to duplicate the cell number after 48 h of HEK293T cell culture, serum from the patient did not increase the cell count.

### 4.4. Soluble Secreted Enzymes

Proteolytic in vitro assays can be performed following reconstitution studies to study soluble secreted enzymes. Recombinant substrates are first purified and radiolabeled with ^125^I and cleavage reactions are carried out at 37 °C. Afterward, substrate and cleavage products are separated by SDS-PAGE and visualized by autoradiography. Equimolar concentrations of WT and mutant proteins from reconstitution assays can be used to study cleavage in heterozygotes. An example of a soluble secreted enzyme in the GH-IGF1 pathway is pregnancy-associated plasma protein-A2 (PAPP-A2), which is a serum and tissue protease responsible for proteolysis of IGFBP-3 and IGFBP-5, regulating the bioavailability of IGF1 and IGF2 to their target tissues [91]. Five affected subjects from two families presenting moderate growth retardation and elevated circulating levels of IGF1, IGF2, IGFBP-3, IGFBP-5, and ALS were found to be homozygous for two different variants in the *PAPPA2* gene (p.D643fs25* and p.A1033V) [92]. Transfection of WT and variants of PAPP-A2 in HEK293 cells was performed in order to study the proteolytic activity of the in vitro reconstituted proteins. WIB showed an absence of expression of p.D643fs25* mutant while expression of p.A1033V was reduced compared to WT. While WT PAPP-A2 was able to cleave IGFBP-3 or -5, neither variant cleaved these substrates, indicating that both mutant PAPP-A2 proteins are enzymatically inactive.

## 5. In Vivo Functional Studies

When studying a particular gene, in vivo studies are essential to validate data collected from in vitro experiments. The most commonly used animal models for in vivo studies are rodents, and mice (*Mus musculus*) in particular. Mice have many advantages over other animal models, thus becoming the most used animal model for studying human biology and disease. Readily available and well-known molecular techniques for genetic manipulation and the animal’s small size facilitates large scale/high throughput studies making it a cost-efficient model. The development of homologous recombination in embryonic stem cells [93,94] by using specific targets to disrupt gene sequences allowed the generation of null mutants, where a gene is disrupted by the introduction of a cassette carrying a positive selection marker, such as the neomycin resistance gene, under the control of a strong promoter [95,96]. It became apparent that, even considering the anatomic and physiological differences between rodents and humans, *GHR* single-gene-KO mice may recapitulate some of the consequences of the lack of GH action (GH-insensitivity mice) [97,98].

To further dissect the impact of the ablation of a gene in a specific tissue, Sauer and Henderson developed the Cre/LoxP system [99], in which a targeted gene was flanked by two loxP sequences (a 34-base pair sequence) that are specifically recognized by Cre, a recombinase protein encoded by the coliphage P1. Recombination occurs specifically at the loxP sequences with loss of the sequence flanked by these two sites. While the gene of interest is flanked by loxP sites, a Cre protein is transfected by using a vector under the control of a tissue-specific enhancer–promoter (for example, the albumin promoter to selectively disrupt gene expression in the liver). With this strategy, the expression of IGF1 was selectively disrupted in the liver by the Cre-mediated site-specific recombination. This was a remarkable achievement that allowed the characterization of the impact of circulating IGF1 on postnatal growth [100].

Another commonly used animal model is the zebrafish (*Danio rerio*) since it has several advantages over other models. Zebrafish are vertebrates and share a high degree of genetic and anatomical homology with humans. Zebrafish spawn and produce large numbers of eggs daily making them ideal for large-scale screening studies. They develop very fast and are optically transparent during embryogenesis allowing the study of early developmental processes for example in embryonic lethal mutants. They are easy to manipulate genetically and also to expose embryos and larvae to various doses of chemicals and observe and score morphological and physiological changes.

While the use of mouse models of a human genetic condition with onset during adulthood is preferred, zebrafish models are ideal for studying pediatric genetic conditions due to their characteristics, which allow the study of early development (such as their optical transparency and rapid development). In addition to making Kos in order to study the role of a particular gene in an animal model like mice or zebrafish, the zebrafish has the potential of being used as a biosensor system to determine the pathogenicity of a given variant suspected of being the cause of a human genetic disease by designing rescue and overexpression assays with which to test those variants and compare them to WT. In order to develop a biosensor system using zebrafish embryos, rescue and overexpression assays are performed. Rescue experiments rely on the use of morpholinos (MOs) to knock down the endogenous zebrafish gene of interest and obtain a scorable phenotype (usually based on morphology). Human WT mRNA is then co-injected with the MO to “rescue” the phenotype. Human mutant mRNA is also co-injected with the MO and the ability to rescue the phenotype is compared to that of WT mRNA. Those variants that are able to rescue the phenotype like WT mRNA are considered benign variants. Variants that are not able to rescue the phenotype are considered pathogenic and hypomorphs are located in between the two [101]. Overexpression assays are based on the ability of human WT mRNA to lead to a scorable phenotype when overexpressed in zebrafish embryos. Only then, human mutant mRNA can be overexpressed and compared to WT. Benign variants will give rise to a phenotype just like WT mRNA, while functionally null variants will lead to no phenotype when overexpressed. Dominant negative variants are suspected, when during a rescue assay, the co-injection of MO and mutant mRNA leads to a more severe phenotype than the injection of MO alone. An overexpression assay can then be performed to confirm the nature of the variant. While human WT mRNA leads to no phenotype, dominant negative mutations will lead to a phenotype when overexpressed [102].

### 5.1. Membrane-Bound Receptors

To date, at least 21 distinct tissue-specific GHR gene disrupted mouse lines have been generated: liver, muscle, fat, brain, bone, heart, intestine, macrophage, pancreatic β cells, hematopoietic stem cells, and multi-tissue “global” [103,104].

Although there is no known zebrafish KO model of GHR, there are several transgenic models of GH, especially fish overexpressing GH or GHR either in the whole organism or in a tissue-specific manner mostly to improve growth for aquaculture [105,106,107]. On the other hand, the mutant *vizzini* with an early stop codon in the *gh1* gene leads to progressively retarded growth in mutants compared to WT animals [108].

### 5.2. Intracellular Transcription Factors

In 1997, *Stat5b* targeted gene disruption in mice led to the *Stat5b*^−/−^ mice [109]. Male-characteristic body growth rates and male-specific liver gene expression were decreased to WT female levels in *Stat5b*^−/−^ males, while female-predominant liver gene products were increased to a level intermediate between WT male and female levels.

On the other hand, in the zebrafish, knockout of *stat5.1,* the ortholog of *STAT5B* in mammals, leads to a significant reduction in body length and body weight in embryos and adults compared to WT fish [110]. The role of this gene appears to be conserved in vertebrates allowing the use of this model to study the pathogenicity of newly identified *STAT5B* gene variants in humans with short stature and immune dysregulation.

Gene targeting of *Stat3* to produce *Stat3* deficient mice led to early embryonic lethality [111], in which case it is very difficult to study the specific role of this gene during development. Tissue-specific disruption of Stat3 in mice hematopoietic cells causes Crohn’s disease-like immunodeficiency [112]. Instead, *Stat3* knock-in mice harboring mutations such as p.Ala727Ser (SA) substitution were produced to study the effect of reduced STAT3 activity [113]. While homozygous mutant mice appeared normal, 75% perinatal lethality was observed in SA/- mice, and the remaining survivors had slow pre- and perinatal growth and altered IGF1 levels. On the other hand, taking advantage of the zebrafish to study genes that lead to early lethality in mice null mutants, morpholino knockdown of *stat3* showed that it is required for planar cell polarity and gastrulation movements during development [114,115]. In addition, null *stat3* zebrafish embryos generated using the transcription activator-like effector nuclease (TALEN) method were viable and completed embryogenesis, but died during juvenile stages exhibiting scoliosis and excessive inflammation [116].

### 5.3. Soluble Secreted Proteins

The ALS knockout mouse or ALS-KO mouse has an inactivation of the *Igfals* gene and surprisingly shows only a mild growth impairment of 13–20% at 10 weeks of age, despite a marked reduction in circulating levels of IGF1 and IGFBP-3 [117]. This mouse model supports the hypothesis that locally produced IGF1 can compensate, at least partially, the decreased levels of circulating IGF1, resulting in the mild growth phenotype observed. Interestingly, overexpression of ALS in mice also caused 13% growth retardation in 4- and 8-week-old mice [118] showing that both the absence and very high levels of ALS lead to the same phenotype since IGF1 is either unprotected from proteolytic attack of IGFBP-3 proteases or sequestered into ternary complexes. Knockdown studies performed in zebrafish embryos using MOs showed that embryos lacking *igfals* resulted in dorsalization while overexpression of either zebrafish *igfals* mRNA or human *IGFALS* mRNA led to ventralization implicating a role for ALS in dorso-ventral patterning during development [119]. This knockdown model will be useful in the future to study the pathogenicity of human variants identified in ALS in short-statured children.

Mouse homozygous for a disruption of the *IGF1* gene (IGF1-KO) show marked growth retardation in utero and postnatally [120,121]. On the other hand, to determine whether circulating vs. autocrine/paracrine IGF1 is critical in postnatal growth, a conditional gene deletion technique using the Cre/loxP system was used to create a liver-specific *IGF1* gene deletion (LID mouse) [100]. While LID mice exhibited normal growth and development, LID mice on a different genetic background revealed a mild decrease of 6% in linear growth [122]. Both, full and conditional mouse Kos, allow for the possibility to study specific human *IGF1* variants by making the corresponding knock-in model. For example, to specifically study the in vivo effect of a variant of interest by the knock-in technique, the WT *Igf1* gene was replaced by the gene encoding mutant *Igf1* with no affinity for IGFBPs (R3-IGF1 and Des-IGF1) expressed under the endogenous *Igf1* promoter in the mouse [123]. Both mutant mouse models presenting reduced serum IGF1 levels showed increased body weight, increased body and bone length, and relative lean mass. They also presented selective organomegaly (spleen, kidneys, and uterus). This result clearly demonstrated the regulatory role of the IGFBPs to establish normal body and organ size.

The only study that used MOs to knockdown *igf1* so far in zebrafish led to embryonic abnormalities that were not possible to discern from nonspecific toxic effects from the MO itself [124].

### 5.4. Soluble Secreted Enzymes

Targeted disruption of *Papp-a2* in mice led to postnatal growth retardation with increased levels of total IGF1 and low levels of free IGF1 [125]. Interestingly, a knock-in mouse model harboring a specific human *PAPP-A2* variant was developed recently [126]. The A1033V-PAPP-A2 variant led to a significant reduction in body length, body weight, relative lean mass and absence of protease activity in mice, which closely resembles the phenotype in humans. Furthermore, zebrafish knockdown of *papp-a2* led to a reduction in cranial cartilages and angiogenesis defects [127]. This zebrafish model will also allow its use as a biosensor assay to determine the pathogenicity of *PAPP-A2* variants identified in humans.

## 6. Conclusions

The rapid technological development of NGS, particularly WES, has resulted in the characterization of several genetic conditions affecting growth and development. Nevertheless, due to the large number of genetic variants identified in a single subject under study, filtering requires the intensive use of bioinformatic tools such as population and disease databases and computational predictive programs to select the most likely variant(s) responsible for the observed phenotype. This preliminary selection often requires further in vitro—and sometimes also in vivo—functional assays, to unequivocally determine the pathogenicity of a novel genetic variant. This long, time-consuming, and very expensive process is considered, in the end, the only scientifically proven method to determine causality between a genetic variant and a human disease.

## Data Availability

Not applicable.

## Supplementary Materials

The following are available online at https://www.mdpi.com/article/10.3390/cells10082063/s1, Table S1: In silico predictive algorithms frequently used for variant pathogenicity inference and for CNV de-tection using NGS data, Table S2: Frequently used population, disease, sequence, and expression databases and Web based useful resources.
