# Supplementary material for: Applying Bioinformatic Platforms, In Vitro, and In Vivo Functional Assays in the Characterization of Genetic Variants in the GH/IGF Pathway Affecting Growth and Development
Source: Cells. 2021 Aug 12;10(8):2063. doi: 10.3390/cells10082063 (PMC8392544; doi:10.3390/cells10082063)
Supplement: Supplementary file 1 [file cells-10-02063-s001.zip › cells-1315123-supplementary.pdf]

cells

Table S1. In silico predictive algorithms frequently used for variant pathogenicity inference and for CNV detection using NGS data[1] [1–5].

Category	Predictor	Website	Based on	Ref.
Nonsynonymous SNVs, particularly missense (small deletions and insertions may be analyzed, depending on predictor)	ConSurf	https://consurf.tau.ac.il/	Evolutionary conservation	6
	FATHMM	http://fathmm.biocom-pute.org.uk		7
	Mutation Assessor	http://mutationassessor.org		8
	PANTHER	http://www.pan-therdb.org/tools/csnp-ScoreForm.jsp		9
	PhD-SNP	http://snps.biofold.org/phd-snp/phd-snp.html		10
	SIFT	https://sift.bii.a-star.edu.sg/		11
	SNPs&GO	http://snps-and-go.bio-comp.unibo.it/snps-and-go	Protein structure/function	12
	Align GVGD	http://agvgd.hci.utah.edu/agvgd_input.php	Protein structure/ function and evolutionary conservation	13
	MutationTaster	http://www.mutationtaster.org		14
	MutPred	http://mutpred.mutdb.org		15
	PolyPhen-2	http://genetics.bwh.har-vard.edu/pph2		16
	LRT	http://www.genet-ics.wustl.edu/jflab/lrt_query.html		17
	VEST3	https://sites.google.com/site/jpopgen/dbNSFP	Functionality	18
	PROVEAN	http://provean.jcvi.org/in-dex.php	Alignment	19
Splicing	GeneSplicer	http://www.cbcb.umd.edu/software/GeneSplicer/gene_spl.shtml	Markov models	20
	Human Splicing Finder	http://www.umd.be/HSF/	Position-dependent logic	21
	MaxEntScan	http://holly-wood.mit.edu/burge-lab/maxent/Xmaxent.html	Maximum entropy principle	22
	NetGene2	http://www.cbs.dtu.dk/ser-vices/NetGene2	Neural networks	23
	NNSplice	http://www.fruit-fly.org/seq_tools/splice.html	Neural networks	24
	FSPLICE	http://www.soft-berry.com/berry.phtml?topic=fsplice&group=programs&sub-group=gfind	Weight matrices (Species-spe-cific)	
	SPANR	http://tools.genes.toronto.edu/	Machine learning	25

Nucleotide conservation	GERP/GERP++	http://mendel.stanford.edu/sidowlab/downloads/gerp/index.html	Genomic evolutionary rate profiling	26, 27
	PhastCons	http://compgen.bscb.cornell.edu/phast/	Conservation scoring	28
	PhyloP	http://compgen.bscb.cornell.edu/phast/help-pages/phyloP.txt	Alignment and phylogenetic trees	29
	SiPhy	https://sites.google.com/site/jpopgen/dbNSFP	Conservation scoring	30
Metapredictors	Condel	http://bg.upf.edu/fannsdb/	Mutation Assessor/ FatHMM	31
	CADD	http://cadd.gs.washington.edu	63 annotations<br>Ex.: GERP/ phastCons/ phyloP / functional genomic / transcript information / protein-level scores (SIFT, Grantham, PolyPhen)	32
	DANN	https://cbcl.ics.uci.edu/public_data/DANN/	Same feature set and training data as CADD but different algorithm for classification	33
	Eigen	https://sites.google.com/site/jpopgen/dbNSFP	SIFT/ PolyPhen/ GERP/ Mutation Assessor/ PhyloP/ PhastCons/ Allele freq	34
	M-CAP	http://bejerano.stanford.edu/mcap/	9 scores + 7 conservation measures<br>SIFT/PolyPhen2/CADD/MutationTaster/Mutation Assessor/FATHMM/ MetaLR/LRT/MetaSVM/PhyloP/ PhastCons/ PAM250/ BLOSUM62/<br>SIPHY/ GERP/ RVIS	35
	MetaLR	https://sites.google.com/site/jpopgen/dbNSFP	10 component scores<br>SIFT/PolyPhen-2 HDIV/ PolyPhen-2 HVAR/ GERP++/Mutation Taster/ Mutation Assessor/ FATHMM/ LRT/ SiPhy/ PhyloP	36
	MetaSVM	https://sites.google.com/site/jpopgen/dbNSFP	10 component scores<br>SIFT/PolyPhen-2 HDIV/ PolyPhen-2 HVAR/ GERP++/Mutation Taster/ Mutation Assessor/ FATHMM/ LRT/ SiPhy/ PhyloP	37
	REVEL	https://sites.google.com/site/revelgenomics	18 scores - 13 tools MutPred/VEST/FATHMM/Polyphen2/SIFT/PROVEAN/MutationAssessor/MutationTaster/LRT/GERP/SiPhy/phyloP/phastCons	38
NMD	NMDescPredictor	https://nmdprediction.shinyapps.io/nmdescpredictor		39

CNV	DECoN	https://github.com/Rahman-Team/DECoN	Depth of coverage.	40
	panelcn.MOPS	https://github.com/bioinf-jku/panelcn.mops	Depth of coverage.	41
	ExomeDepth	https://github.com/vplagnol/ExomeDepth	Depth of coverage.	42
	CoNVaDING	https://github.com/mol-genis/CoNVaDING	Depth of coverage.	43
	CODEX2	https://github.com/yuchaojiang/CODEX2	Depth of coverage.	44
	BreakDancer	http://breakdancer.source-forge.net/	Paired-end mapping	45
	PEMer	http://sv.gerstein-lab.org/pemer/	Paired-end mapping	46
	Ulysses	https://github.com/gillet/ulys-ses	Paired-end mapping	47
	PRISM	http://compbio.cs.to-ronto.edu/prism/	Split-reads	48
	Gustaf	http://www.seqan.de/pro-jects/gustaf/	Split-reads	49
	Pindel	http://gmt.ge-nome.wustl.edu/packages/pin-del/	Split-reads	50
	Magnolya	http://bioinformat-ics.tudelft.nl/dbl/software	Assembly	51

1. Richards, S.; Aziz, N.; Bale, S.; Bick, D.; Das, S.; Gastier-Foster, J.; Grody, W.W.; Hegde, M.; Lyon, E.; Spector, E.; Voelkerding, K.; Rehm, H. L.; ACMG Laboratory Quality Assurance Committee. Standards and guidelines for the interpretation of sequence variants: a joint consensus recommendation of the American College of Medical Genetics and Genomics and the Association for Molecular Pathology. Genet. Med. 2015, 17, 405-424. doi: 10.1038/gim.2015.30.
2. Ghosh, R.; Oak, N.; Plon, S. E. Evaluation of in silico algorithms for use with ACMG/AMP clinical variant interpretation guidelines. Genome Biol. 2017, 18, 225. doi: 10.1186/s13059-017-1353-5.
3. Moreno-Cabrera, J. M.; Del Valle, J.; Castellanos, E..; Feliubadaló, L.; Pineda, M.; Brunet, J.; Serra, E.; Capellà, G.; Lázaro, C.; Gel, B. Evaluation of CNV detection tools for NGS panel

data in genetic diagnostics. Europ. J. Hum. Genet. 2020, 28, 1645–1655. doi.org/10.1038/s41431-020-0675-z).

4. Pirooznia, M.; Goes, F. S.; Zandi, P. P. Whole-genome CNV analysis: advances in computational approaches. Front. Genet. 2015, 138. https://doi.org/10.3389/fgene.2015.00138),
5. Teo, S. M.; Pawitan, Y.; Ku, C. S.; Chia, K. S.; Salim, A. (2012). Statistical challenges associated with detecting copy number variations with next-generation sequencing. Bioinformatics 2012 28, 2711–2718. https://doi.org/10.1093/bioinformatics/bts535)
6. Ashkenazy, H.; Abadi, S.; Martz, E.; Chay, O.; Mayrose, I.; Pupko, T.; Ben-Tal, N. ConSurf 2016: an improved methodology to estimate and visualize evolutionary conservation in macromolecules. *Nucleic Acids Res.* **2016,** 44, W344–W350. doi: 10.1093/nar/gkw408.
7. Shihab, H.A.; Gough, J.; Cooper, D.N.; Stenson, P.D.; Barker, G.L.; Edwards, K.J.; Day, I.N.; Gaunt, T.R. Predicting the functional, molecular, and phenotypic consequences of amino acid substitutions using hidden Markov models. *Hum. Mutat.* **2013**, 34, 57–65. doi: 10.1002/humu.22225.
8. Reva, B.; Antipin, Y.; Sander, C. Predicting the functional impact of protein mutations: application to cancer genomics. *Nucleic Acids Res*. **2011**, 39, e118. doi: 10.1093/nar/gkr407.
9. Mi, H.; Ebert, D.; Muruganujan, A.; Mills, C.; Albou, L.P.; Mushayamaha, T.; Thomas, P.D. PANTHER version 16: a revised family classification, tree-based classification tool, enhancer regions and extensive API. *Nucleic Acids Res*. **2021**, 49, D394–D403. doi: 10.1093/nar/gkaa1106.
10. Capriotti, E.; Calabrese, R.; Casadio, R. Predicting the insurgence of human genetic diseases associated to single point protein mutations with support vector machines and evolutionary information. *Bioinformatics* **2006**, 22, 2729–2734. doi: 10.1093/bioinformatics/btl423.
11. Sim, N.L.; Kumar, P.; Hu, J.; Henikoff, S.; Schneider, G.; Ng, P.C. SIFT web server: predicting effects of amino acid substitutions on proteins. *Nucleic Acids Res*. **2012,** 40, W452-457. doi: 10.1093/nar/gks539.
12. Calabrese, R.; Capriotti, E.; Fariselli, P.; Martelli, P.L.; Casadio, R. Functional annotations improve the predictive score of human disease-related mutations in proteins. *Hum. Mutat***.** **2009**, 30, 1237-1244. doi: 10.1002/humu.21047.

13. Tavtigian, S.V.; Deffenbaugh, A.M.; Yin, L.; Judkins, T.; Scholl, T.; Samollow, P.B.; de Silva, D.; Zharkikh, A.; Thomas, A. Comprehensive statistical study of 452 BRCA1 missense substitutions with classification of eight recurrent substitutions as neutral. *J. Med. Genet.* **2006,** 43, 295–305. doi: 10.1136/jmg.2005.033878.

14. Schwarz, J. M.; Cooper, D. N.; Schuelke, M.; Seelow, D. MutationTaster2: mutation prediction for the deep-sequencing age. *Nat. Methods* **2014**, 11, 361–362. doi: 10.1038/nmeth.2890.

15. Pejaver, V.; Urresti, J.; Lugo-Martinez, J.; Pagel, K. A.; Lin, G. N.; Nam, H. J.; Mort, M.; Cooper, D. N.; Sebat, J.; Iakoucheva, L. M.; Mooney, S. D.; & Radivojac, P. Inferring the molecular and phenotypic impact of amino acid variants with MutPred2. *Nat. Commun.* **2020,** *11*, 5918. doi: 10.1038/s41467-020-19669-x.

16. Adzhubei, I. A.; Schmidt, S.; Peshkin, L.; Ramensky, V. E.; Gerasimova, A.; Bork, P.; Kondrashov, A. S.; Sunyaev, S. R. A method and server for predicting damaging missense mutations. *Nat. Methods* **2010,** 7, 248–249. doi: 10.1038/nmeth0410-248.

17. Chun, S.; Fay, J. C. Identification of deleterious mutations within three human genomes. *Genome Res.* **2009,** *19*, 1553–1561. doi: 10.1101/gr.092619.109.

18. Carter, H.; Douville, C.; Stenson, P. D.; Cooper, D. N.; Karchin, R. Identifying Mendelian disease genes with the variant effect scoring tool. *BMC Genomics*. **2013,** 14, Suppl 3, S3. doi: 10.1186/1471-2164-14-S3-S3.

19. Choi, Y.; Chan, A. P. PROVEAN web server: a tool to predict the functional effect of amino acid substitutions and indels. *Bioinformatics* **2015,** 31, 2745–2747. doi: 10.1093/bioinformatics/btv195.

20. Pertea, M.; Lin, X.; Salzberg, S. L. GeneSplicer: a new computational method for splice site prediction. *Nucleic Acids Res*. **2001**, 29, 1185–1190. doi: 10.1093/nar/29.5.1185.

21. Desmet, F. O.; Hamroun, D.; Lalande, M.; Collod-Béroud, G.; Claustres, M.; Béroud, C. Human Splicing Finder: an online bioinformatics tool to predict splicing signals. *Nuceic Acids Res.* **2009,** 37, e67. doi: 10.1093/nar/gkp215.

22. Yeo, G.; and Burge, C. B. Maximum entropy modeling of short sequence motifs with applications to RNA splicing signals. *J. Comput. Biol.* **2004**, 11, 377–394. doi: 10.1089/1066527041410418.

23. Hebsgaard, S. M.; Korning, P. G.; Tolstrup, N.; Engelbrecht, J.; Rouzé, P.; Brunak, S. Splice site prediction in Arabidopsis thaliana pre-mRNA by combining local and global sequence information. *Nucleic Acids Res*. **1996,** 24, 3439–3452. doi: 10.1093/nar/24.17.3439.
24. Reese, M. G.; Eeckman, F. H.; Kulp, D.; Haussler, D. Improved splice site detection in Genie. *J. Comput. Biol*. **1997,** *4*, 311–323. doi: 10.1089/cmb.1997.4.311.
25. Xiong, H. Y.; Alipanahi, B.; Lee, L. J.; Bretschneider, H.; Merico, D.; Yuen, R. K. C.;[3], Hua, Y.; Gueroussov, S.;, Najafabadi, H. S.; Hughes, T. R.; Morris, Q.; Barash, Y.; Krainer, A. R.; Jojic, N.; Scherer, S. W.; Blencowe, B. J.; Brendan J Frey, B. J. The human splicing code reveals new insights into the genetic determinants of disease. *Science* **2015**, 347, 1254806. doi: 10.1126/science.1254806.
26. Cooper, G. M.; Stone, E. A.; Asimenos, G.; NISC Comparative Sequencing Program, Green, E. D.; Batzoglou, S.; Sidow, A. Distribution and intensity of constraint in mammalian genomic sequence. *Genome Res***. 2005,** 15, 901–913. doi: 10.1101/gr.3577405.
27. Davydov, E. V.; Goode, D. L.; Sirota, M.; Cooper, G. M.; Sidow, A.; Batzoglou, S. Identifying a high fraction of the human genome to be under selective constraint using GERP++. *PLoS Comput. Biol.* **2010,** 6, e1001025. doi: 10.1371/journal.pcbi.1001025.
28. Hubisz, M. J.; Pollard, K. S.; Siepel, A. PHAST and RPHAST: phylogenetic analysis with space/time models. *Brief. Bioinform.* **2011,** 12, 41–51. doi: 10.1093/bib/bbq072.
29. Pollard, K. S.; Hubisz, M. J.; Rosenbloom, K. R.; Siepel, A: Detection of nonneutral substitution rates on mammalian phylogenies. *Genome Res*. **2010,** 20, 110-121. doi: 10.1101/gr.097857.109.
30. Garber, M.; Guttman, M.; Clamp, M.; Zody, M. C.; Friedman, N.; Xie, X. Identifying novel constrained elements by exploiting biased substitution patterns. *Bioinformatics* **2009,** 25, i54–i62. doi: 10.1093/bioinformatics/btp190.
31. González-Pérez, A.; López-Bigas, N. Improving the assessment of the outcome of nonsynonymous SNVs with a consensus deleteriousness score, Condel. *Am. J. Hum. Genet.* **2011,** 88, 440–449. doi: 10.1016/j.ajhg.2011.03.004.

32. Kircher, M.; Witten, D. M.; Jain, P.; O'Roak, B. J.; Cooper, G. M.; Shendure, J. A general framework for estimating the relative pathogenicity of human genetic variants. *Nat. Gen.* **2014,** 46, 310–315. doi: 10.1038/ng.2892.
33. Quang, D.; Chen, Y.; Xie, X. DANN: a deep learning approach for annotating the pathogenicity of genetic variants. *Bioinformatics* **2015,** 31, 761–763. oi: 10.1093/bioinformatics/btu703.
34. Ionita-Laza, I.; McCallum, K.; Xu, B.; Buxbaum, J. D. A spectral approach integrating functional genomic annotations for coding and noncoding variants. *Nat. Genet*. **2016,** 48, 214-220. doi: 10.1038/ng.3477.
35. Jagadeesh, K. A.; Wenger, A. M.; Berger, M. J.; Guturu, H.; Stenson, P. D.; Cooper, D. N.; Bernstein, J. A.; Bejerano, G. M-CAP eliminates a majority of variants of uncertain significance in clinical exomes at high sensitivity. *Nat. Gen*. **2016,** 48, 1581–1586. doi: 10.1038/ng.3703.
36. Dong, C.; Wei, P.; Jian, X.; Gibbs, R.; Boerwinkle, E.; Wang, K.; Liu, X. Comparison and integration of deleteriousness prediction methods for nonsynonymous SNVs in whole exome sequencing studies. *Hum. Mol. Genet*. **2015,** 24, 2125-37. doi: 10.1093/hmg/ddu733.
37. Kim, S.; Jhong, J. H.; Lee, J.; Koo, J. Y. Meta-analytic support vector machine for integrating multiple omics data. *BioData Min*. **2017**, 10, 2. doi: 10.1186/s13040-017-0126-8.
38. Ioannidis, N. M.; Rothstein, J. H.; Pejaver, V.; Middha, S.; McDonnell, S. K.; Baheti, S.; Musolf, A.; Li, Q.; Holzinger, E.; Karyadi, D.; Cannon-Albright, L. A.; Teerlink, C. C.; Stanford, J. L.; Isaacs, W. B.; Xu, J.; Cooney, K. A.; Lange, E. M.; Schleutker, J.; Carpten, J. D.; Powell, I. J.; Cussenot, O.; Cancel-Tassin, G.; Giles, G. G.; MacInnis, R. J.; Maier, C.; Hsieh, C. L.; Wiklund, F.; Catalona, W. J.; Foulkes, W. D.; Mandal, D.; Eeles, R. A.; Kote-Jarai, Z.; Bustamante, C. D.; Schaid, D. J.; Hastie, T.; Ostrander, E. A.; Bailey-Wilson, J. E.; Radivojac, P.; Thibodeau, S. N.; Whittemore, A. S.; Sieh, W. REVEL: An ensemble method for predicting the pathogenicity of rare missense variants. *Am. J. Hum. Genet.* **2016**, 99, 877-885. doi: 10.1016/j.ajhg.2016.08.016.

39. Hsu, M.K.; Lin H.Y.; Chen, F.C. NMD Classifier: A reliable and systematic classification tool for nonsense-mediated decay events. *Plos One* **2017**, 12, e0174798. doi: 10.1371/journal.pone.0174798.
40. Fowler, A.; Mahamdallie, S.; Ruark, E.; Seal, S.; Ramsay, E.; Clarke, M.; Uddin, I.; Wylie, H.; Strydom, A.; Lunter, G.; Rahman, N. Accurate clinical detection of exon copy number variants in a targeted NGS panel using DECoN. *Wellcome Open Res*. **2016**, 1, 20. doi: 10.12688/wellcomeopenres.10069.1.
41. Povysil, G.; Tzika, A.; Vogt, J.; Haunschmid, V.; Messiaen, L.; Zschocke, J.; Klambauer, G.; Hochreiter, S.; Wimmer, K. panelcn.MOPS: Copy-number detection in targeted NGS panel data for clinical diagnostics. *Hum. Mut.* **2017,** *38*, 889–897. doi: 10.1002/humu.23237.
42. Plagnol, V.; Curtis, J.; Epstein, M.; Mok, K. Y.; Stebbings, E.; Grigoriadou, S.; Wood, N. W.; Hambleton, S.; Burns, S. O.; Thrasher, A. J.; Kumararatne, D.; Doffinger, R.; Nejentsev, S. A robust model for read count data in exome sequencing experiments and implications for copy number variant calling. *Bioinformatics* **2012**, 28, 2747–2754. doi: 10.1093/bioinformatics/bts526.
43. Johansson, L. F.; van Dijk, F.; de Boer, E. N.; van Dijk-Bos, K. K.; Jongbloed, J. D.; van der Hout, A. H.; Westers, H.; Sinke, R. J.; Swertz, M. A.; Sijmons, R. H.; Sikkema-Raddatz, B. CoNVaDING: Single exon variation detection in targeted NGS data. *Hum. Mut.* **2016**, 37, 457–464. doi: 10.1002/humu.22969.
44. Jiang, Y.; Wang, R.; Urrutia, E.; Anastopoulos, I. N.; Nathanson, K. L.; Zhang, N. R. CODEX2: full-spectrum copy number variation detection by high-throughput DNA sequencing. *Genome Biol*. **2018**, *19*, 202. doi: 10.1186/s13059-018-1578-y.
45. Chen, K.; Wallis, J.; McLellan, M.; Larson, D. E.; Kalicki, J. M.; Pohl, C. S.; McGrath, S. D.; Wendl, M. C.; Zhang, Q.; Locke, D. P.; Shi, X.; Fulton, R. S.; Ley, T. J.; Wilson,R. K.; Ding, L.; Mardis, E. R. BreakDancer: an algorithm for high-resolution mapping of genomic structural variation. *Nat. Methods.* **2009**, 6, 677–681. doi: 10.1038/nmeth.1363.

46. Korbel, J. O.; Abyzov, A.; Mu, X. J.; Carriero, N.; Cayting, P.; Zhang, Z.; Snyder, M.; Gerstein, M. B. PEMer: a computational framework with simulation-based error models for inferring genomic structural variants from massive paired-end sequencing data. *Genome Biol.* **2009,** 10, R23. doi: 10.1186/gb-2009-10-2-r23.
47. Gillet-Markowska, A.; Richard, H.; Fischer, G.; Lafontaine, I. Ulysses: accurate detection of low-frequency structural variations in large insert-size sequencing libraries. *Bioinformatics* **2015,** *31*, 801–808. doi: 10.1093/bioinformatics/btu730.
48. Jiang, Y.; Wang, Y.; Brudno, M. PRISM: pair-read informed split-read mapping for base-pair level detection of insertion, deletion and structural variants. *Bioinformatics* 2012**,** 28, 2576–2583. doi: 10.1093/bioinformatics/bts484.
49. Trappe, K.; Emde, A. K.; Ehrlich, H. C.; Reinert, K. Gustaf: Detecting and correctly classifying SVs in the NGS twilight zone. *Bioinformatics* **2014**, 30, 3484–3490. doi: 10.1093/bioinformatics/btu431.
50. Ye, K.; Schulz, M. H.; Long, Q.; Apweiler, R.; Ning, Z. Pindel: a pattern growth approach to detect break points of large deletions and medium sized insertions from paired-end short reads. *Bioinformatics* **2009**, 25, 2865–2871. doi: 10.1093/bioinformatics/btp394.
51. Nijkamp, J. F.; van den Broek, M. A.; Geertman, J. M.; Reinders, M. J.; Daran, J. M.; de Ridder, D. De novo detection of copy number variation by co-assembly. *Bioinformatics* **2012**, 28, 3195–3202. doi: 10.1093/bioinformatics/bts601.

Table S2. Frequently used population, disease, sequence, and expression databases and Web based useful resources.

**Population databases**		
**gnomAD**	The Genome Aggregation Database (gnomAD) is a resource developed by an international coalition of investigators, with the goal of aggregating and harmonizing both exome and genome sequencing data from a wide variety of large-scale sequencing projects, and making summary data available for the wider scientific community.	https://gnomad.broadinstitute.org/
**EVS**	The goal of the NHLBI GO Exome Sequencing Project (ESP), Exome Variant Server (**EVS**),_ is to discover novel genes and mechanisms contributing to heart, lung and blood disorders by pioneering the application of next-generation sequencing of the protein coding regions of the human genome across diverse, richly-phenotyped populations and to share these datasets and findings with the scientific community to extend and enrich the diagnosis, management and treatment of heart, lung and blood	https://evs.gs.washington.edu/EVS/
**dbSNP**	Single nucleotide polymorphism database (**dbSNP**) contains human single nucleotide variations, microsatellites, and small-scale insertions and deletions along with publication, population frequency, molecular consequences, and genomic and RefSeq mapping information for both common variations and clinical mutations.	https://www.ncbi.nlm.nih.gov/snp/?cmd=search
**dbVar**	Variation database (**dbVar**) is NCBI's database of human genomic Structural Variation – large variants >50 bp – including insertions, deletions, duplications, inversions, mobile elements, translocations, and complex variants.	https://www.ncbi.nlm.nih.gov/dbvar/
**Disease databases**		
**ClinVar**	Clinical Variation (ClinVar) aggregates information about genomic variation and its relationship to human health.	https://www.ncbi.nlm.nih.gov/clinvar/
**OMIM**	Online Mendelian Inheritance in Man (OMIM) is an online catalog of human genes and genetic disorders.	https://www.omim.org/

**HGMD**	The Human Gene Mutation Database (HGMD®) represents an attempt to collate all known (published) gene lesions responsible for human inherited disease.	http://www.hgmd.cf.ac.uk/ac/index.php
**LOVD**	Leiden Open Variation Database (LOVD) Online gene-centered collection and display of DNA variants.	https://www.lovd.nl/
**Sequence databases**		
**NCBI Genome**	This resource organizes information on genomes including sequences, maps, chromosomes, assembles, and annotations.	https://www.ncbi.nlm.nih.gov/genome/
**RefSeqGene**	This database includes genomic sequences, location and number of exons, sequence(s) of the reference cDNA(s), and sequence(s) of the protein product(s).	https://www.ncbi.nlm.nih.gov/refseq/rsg/
**LRG**	The Locus Reference Genomic (LRG) record contains stable reference sequences that are used for reporting sequence variants with clinical implications.	https://www.lrg-sequence.org/
**MitoMap**	A human mitochondrial genome database.	https://www.mitomap.org/MITOMAP
**Ensembl**	Genome browser for vertebrate genomes that supports research in comparative genomics, evolution, sequence variation and transcriptional regulation. Ensembl annotate genes, computes multiple alignments, predicts regulatory function and collects disease data.	http://www.ensembl.org/
**Expression databases (mRNA and protein)**		
**GTEx**	Genotype-Tissue Expression Portal: resource to study human gene expression and regulation and its relationship to genetic variation	https://www.gtexportal.org/home/
**The Human Protein Atlas**	Open access Swedish-based program to allow scientists to access the data for exploration of the human proteome. Aim: to map all the human proteins in cells, tissues and organs using an integration of various omics technologies.	https://www.proteinatlas.org/
**Web based useful resources**		
**ClinGen**	ClinGen is a National Institutes of Health (NIH)-funded resource dedicated to building an authoritative central resource that defines the clinical relevance of genes and variants for use in precision medicine and research.	https://clinicalgenome.org/
**Genomics England PanelApp**	Publicly-available knowledgebase that allows virtual gene panels related to human disorders to be created, stored and queried.	https://panelapp.genomicsengland.co.uk/

**VarSome**	VarSome.com is a community-driven project aimed at sharing global expertise on human variants.	https://varsome.com/
**FRANKLIN**	Community version of Artificial Intelligence-Based Variant Classification Engine	https://franklin.genoox.com/clinical-db/home
**MARRVEL**	MARRVEL (Model organism Aggregated Resources for Rare Variant ExpLoration) aims to facilitate the use of public genetic resources to prioritize rare human gene variants for study in model organisms. The key biological and genetic features are then extracted from existing model organism databases (SGD, PomBase, WormBase, FlyBase, ZFIN, MGI, and RGD).	http://marrvel.org/
**Mastermind Genomic Search Engine**	Comprehensive search and association engine to identify gene, variant, disease, phenotype, and therapy evidence from scientific articles.	https://mastermind.genomenon.com/
**Mutalyzer**	Program designed to automatically apply the Human Genome Variation Society guidelines for sequence variant nomenclature.	https://www.mutalyzer.nl/
